# Gender and Age-Specific Differences in the Association of Thyroid Function and Hyperuricemia in Chinese: A Cross-Sectional Study

**DOI:** 10.1155/2022/2168039

**Published:** 2022-07-05

**Authors:** Ming Yang, Suyan Cao

**Affiliations:** VIP Department, General Medicine Department and Health Management Centre, Beijing Hospital, National Center of Gerontology, Institute of Geriatric Medicine, Chinese Academy of Medical Sciences, No. 1 Da Hua Road, Dongcheng District, Beijing 100037, China

## Abstract

**Objective:**

We aimed to explore gender and age-specific influences on the association between thyroid function and hyperuricemia (HUA) in a large Chinese population.

**Methods:**

A total of 19,013 individuals (10,563 males and 8,450 females) were recruited. The association between HUA and thyroid function was analyzed by multivariate logistic regression, and the analyses were stratified by gender and age. Thyroid function subgroups were determined in 2 methods including thyroid status and thyroid-stimulating hormone (TSH) quartiles.

**Results:**

Overall prevalence of serum uric acid (SUA) and HUA was significantly higher in males, while TSH value and thyroid dysfunction were higher in females. Increasing trends of the TSH level in both genders as well as HUA prevalence in females were found positively along with aging. However, males showed a reduced trend in HUA risk negatively with aging. Our population showed that the risk of developing HUA in hyperthyroidism, normal euthyroidism, mild hypothyroidism, and overt hypothyroidism subgroups had adjusted ORs of 0.634, 1.229, 1.370, and 1.408, respectively, in males. Subjects in females showed a similar increased risk of HUA with ORs of 0.770, 1.198, 1.256, and 1.458, respectively. Similar tendency was observed in TSH quartiles; the above two models showed significantly higher risk of HUA in the high TSH group of males, but not of females. Aging was a significant risk factor for HUA, particularly in older females after adjusting for TSH.

**Conclusion:**

The risk of HUA was positively associated with an elevation in TSH levels in both genders irrespective of age, indicating the protective effects of low TSH on HUA. Males with high TSH value were more vulnerable to suffer significant risk of HUA.

## 1. Introduction

It is well known that hyperuricemia (HUA) is a common metabolic disorder, and its incidence is increasing along with the years [1]. Previous epidemiological studies proved that HUA is associated with components of metabolic syndrome, such as hypertension, diabetes, insulin resistance, and dyslipidemia. Physical activity, current smoking, drinking, and sedentary lifestyle contributed to occurrence of HUA [2, 3]. A meta-analysis covering the years from 2000 to 2014 reported the prevalence of HUA in mainland China was 13.3% (19.4% in men and 7.9% in women) according to the data from different regions [4].

Recently, the available limited literature showed that a different thyroid function was correlated with the prevalence of HUA and displayed with conflicting results [5–9]. Gender and age can influence the relationship between thyroid function and HUA [10, 11]. However, information about the association between HUA and thyroid function in Chinese population with the effect of gender and age-stratified is scarce. The aim of the current study was to evaluate the gender and age-specific differences in the association between thyroid dysfunction and HUA in a large cross-sectional Chinese population from the epidemiological aspect.

## 2. Subjects and Methods

### 2.1. Design

**Figure 1:**
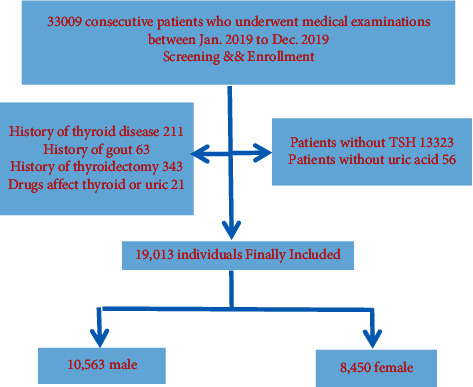
Patient flow chart. TSH,= thyrotropin.

This cross-sectional, community-based health check study was carried out in the health examination center of Beijing Hospital in China, under collaborative efforts from the Department of Health Management. During the period from January 2019 to December 2019, a total of 19,013 eligible individuals (10,563 males and 8,450 females) who underwent medical examinations at our institution were consecutively enrolled (Figure 1). The participants were identified using the following exclusion criteria: subjects with a disease history of the thyroid, gout, and thyroidectomy; subjects with any disease or taking any medicine that might affect the thyroid or uric acid (e.g., antithyroid drugs, thyroid hormone, amiodarone, iodine, estrogen, androgen, statins, and steroid hormones); and patients without available data on thyroid function and uric acid. The study was approved by the Institutional Review Board and Ethics Committee of Beijing Hospital.

### 2.2. Measurements

Each participant received anthropometric measurements and fasting blood tests in our institution. Venous blood samples after overnight fasting were taken in the morning and analyzed within an hour in the hospital. Physical examination including body height (BH), body weight (BW), waist circumference (WC), systolic blood pressure (SBP), and diastolic blood pressure (DBP) were measured using standardized methods. Body mass index (BMI) was calculated as BW divided by BH squared (kg/m^2^). Biochemical data including serum uric acid (SUA), creatinine (Cr), blood urea nitrogen (BUN), fasting glucose (FG), triglycerides (TG), total cholesterol (TC), low-density lipoprotein cholesterol (LDL-C), alanine aminotransferase (ALT), and aspartate aminotransferase (AST) were measured enzymatically by an autoanalyzer (Hitachi Model 7170 analyzer, Hitachi, Tokyo, Japan). Serum thyroid-stimulating hormone (TSH), total thyroxine (TT4), and total triiodothyronine (TT3) levels were analyzed on an automated ADVIA Centaur analyzer (Siemens Healthcare Diagnostics, New York, USA) by the chemiluminescent reaction principle. Thyroglobulin autoantibodies (TgAb) were measured by a specific IRMA assay (TG-Ab IRMA; Biocode) and thyroid peroxidase autoantibodies (TPOAb) were measured by a specific RIA (AB-TPO; Sorin Biomedica). Finally, we calculated eGfr of each patient's group in both sexes. The modified equation by Chinese investigators was used: e-GFR = 186 × (serum creatinine−1.154) × (age−0.203) × 1.233 (if Chinese) × 0.742 (if female).

### 2.3. Definitions

The laboratory reference ranges for the parameters were as follows: TSH 0.35–5.5 mIU/mL; TT3 3.5–6.5 pmol/L; TT4 11.5–23.5 pmol/L; TPOAb <70 IU/mL; and TgAb <70 IU/mL. Thyroid function subgroups were determined in 2 methods. Five grades of thyroid functional status were determined. Grade I: perfect euthyroidism (TSH from 0.35 to 2.5 *μ*IU/mL, normal TT3 and TT4); grade II: normal euthyroidism (TSH from 2.5 to 5.5 *μ*IU/mL, normal TT3 and TT4); grade III: mild hypothyroidism (TSH from 5.5 to 10.0 *μ*IU/mL); grade IV: overt hypothyroidism (TSH greater than 10.0 *μ*IU/mL); and grade V: hyperthyroidism (TSH less than 0.35 *μ*IU/mL). In the second method, serum TSH values were divided based on the quartiles of the measurements. Hyperuricemia was defined as SUA >420 *μ*mol/L (7.0 mg/dL) in men and SUA >360 *μ*mol/L (6.0 mg/dL) in women.

### 2.4. Statistical Analysis

Data were generally analyzed based on different genders. Continuous variables with normal distributions (age, BMI, WC, SBP, DBP, TSH, TT3, TT4, TC, FG, BUN, Cr, UA, FG, ALT, AST, and glomerular filtration rate (GFR)) were expressed as mean ± standard deviation. Their intergroup differences were tested by using the *t*-test or one-way analysis of variance. The remaining variables (TG and hsCRP) with skewed distributions were expressed as median (interquartile range (IQR)). Their intergroup differences were tested by using the Mann–Whitney *U* test. The chi-squared test was used to compare intergroup prevalence differences. Pearson bivariate correlation was made between SUA and other variables. By stratifying the data by thyroid function, the odds ratio (OR) for HUA with 95% confidence interval (CI) was calculated by the binary logistic regression model. Statistical Package for Social Sciences (SPSS version 17.0, Chicago, IL, USA) was used to conduct statistics, and significance was defined as *P* < 0.05.

## 3. Results

### 3.1. Characteristics of the Participants in Different Genders

The demographic characteristics, anthropometric measurements, and biochemical features of the participants were summarized (Table 1). The enrolled 19,013 population comprised of 10,563 (58.25%) males and 8,450 (41.75%) females. The age ranged from 16 to 97 years, with a mean age of 47.50 ± 14.51 years. Males were older than females; BMI, WC, SBP, and DBP in males were higher. In terms of serum lipids, TC was lower in males, yet LDL was higher in males. Besides, hepatic function indicators like ALT and AST were higher in males than in females. Furthermore, renal function like GFR was lower in males; Cr and BNU levels were higher in males. Overall prevalence of HUA was 17.4%, with 25.0% in males and 7.9% in females. The median UA concentrations in males and females were 372.81 *μ*mol/L and 272.28 *μ*mol/L, respectively, significantly higher in men. TSH and TT4 were significantly lower, while TT3 was higher in males than in females. Of the total subjects, 693 (3.6%) were positive for TgAb, 783 (4.1%) were positive for TPOAb, and the prevalence of elevated serum thyroid autoantibodies was significantly higher among women than in men.

### 3.2. Prevalence of Hyperuricemia in Different Genders

**Figure 2:**
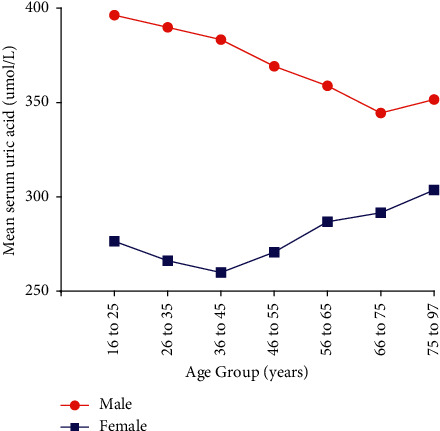
Mean serum uric acid in different age subgroups.

**Figure 3:**
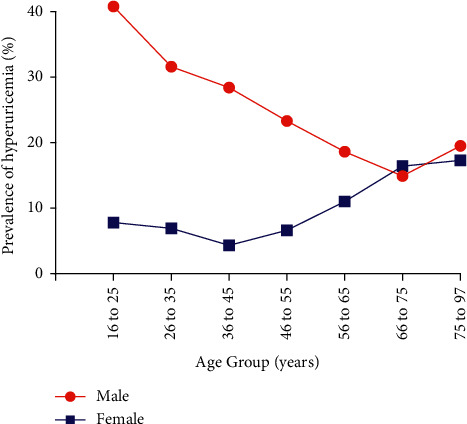
Prevalence of hyperuricemia in different age subgroups.

The differences of SUA and HUA based on age groups are shown in Figures 2 and 3. The prevalence rate of HUA and the mean SUA level showed a U-shaped distribution in terms of age, although it was least prevalent in women aged 36–45 years and men aged 66–75 years. In other words, in women, SUA and HUA slightly decreased with age until the 3rd decade, then greatly increased with age, and reached the highest in the 7th decade. While in men, SUA and HUA were rapidly declined with age until the 6th decade.

The crisscross pattern was discovered in HUA, young males (younger than 55) had significantly higher prevalence than females. However, after menopause (older than 65), females had a significantly higher prevalence than males, yet the converging point was around 66–75 years of age. For SUA, males always showed a higher prevalence than females, irrespective of their age.

### 3.3. Prevalence of Thyroid Function in Different Genders

Our study demonstrated that females had a significantly higher overall incidence rate of thyroid dysfunction than males (Table 2). Splitting the whole cohort into groups of thyroid status, of which 7984 (8450 women, 94.5%) subjects were identified with euthyroidism, 380 (4.5%) with hypothyroidism, and the population with hyperthyroidism had 86 patients, 1.0% in females. While in males, 10240 (10563 men, 96.9%) with euthyroidism, 264 (2.5%) with hypothyroidism, and 59 (0.6%) individuals with hyperthyroidism. The chi-squared test showed significance in hypothyroidism (Chi-square value = 57.254, *p* < 0.01) and in hyperthyroidism (chi-square value = 13.080, *p* < 0.01) between genders. When we analyzed the detailed incidences stratified by age, most age subgroups demonstrated the same pattern of differences between gender, except for the youngest (age≤25 years old) and menopause (age 56–65 years old).

**Figure 4:**
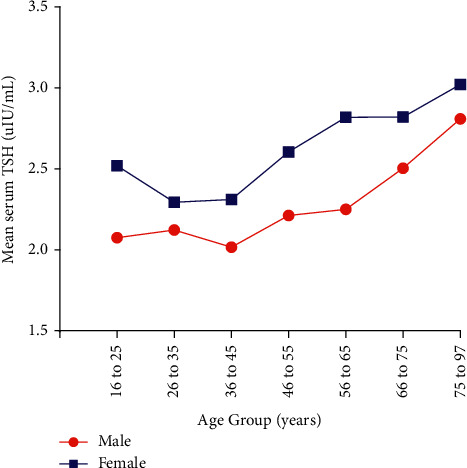
Mean serum TSH in different age subgroups. TSH,= thyrotropin.

Another important finding was that there existed a significant tendency of increasing hypothyroidism rate with aging for both genders. Similarly, by analysis, the presence of TSH level progressively increased with increasing age (Figure 4).

### 3.4. Correlations of UA and Other Parameters in Different Genders

Pearson bivariate correlation showed the association between SUA and other variables (Table 3). The closest relationship of all variables appeared to be between BMI and SUA in both genders. The other top factors belonged to WC, TG, age, and Cr in males and WC, TG, Cr, and BUN in females. Moreover, only the coefficients of BMI exceeded 0.250 in males; yet in women, the coefficients of all top five parameters were higher than 0.270. However, age was observed negatively correlated with SUA in males (−0.183) and positively associated with SUA in females (0.164).

### 3.5. Risks of Developing HUA in Different Genders

Three binary logistic regression models were used to calculate the risks of developing HUA (Table 4).

The 1st model designated the thyroid functional state as categorical variable, and the subgroup of TSH ranging from 0.35 to 2.5 mIU/mL was determined as reference. Age was included as a covariate. This model revealed that normal euthyrodism and mild hypothyroidism were independent risk factors for HUA in males, which showed adjusted ORs of 1.229 (1.107–1.363) and 1.370 (1.006–1.866). However, for females, normal euthyrodism was demonstrated to be a meaningful risk factor of HUA, with an OR of 1.198 (1.011–1.420). Our population in males showed that normal euthyroidism, mild hypothyroidism, and overt hypothyroidism had ORs of 1.229, 1.370, and 1.408, respectively. Subjects in females showed a similar increasing trend with an ORs of 1.198, 1.256, and 1.458, respectively, compared with the reference TSH subgroup risk. Compared with perfect euthyroidism, the risks of the hyperthyroidism group were 0.634 and 0.770 in males and females, respectively; however, both genders did not reach significance. Therefore, we demonstrated significantly reduced risk of HUA, while TSH decreased from the reference level, indicating the protective effects of low TSH on HUA.

The 2nd model designated TSH quartiles as the categorical variables and age as the covariate, and the lowest quartile was set as reference. Significantly increased risk was demonstrated in quartile 4 for males, while for females, no meaningful OR was found in all TSH quartiles. Or the value is steadily becoming higher along with the TSH value increased. For instance, from TSH quartiles 2–4, the ORs were 1.076, 1.108, and 1.322, respectively, in males, and in accordance with the results in women, the ORs were 0.887, 0.976, and 1.119 in females, respectively. Consequently, in the above two models, the risk of HUA was positively associated with an elevation in TSH levels (*p* < 0.001).

The 3rd model designated age subgroups as categorical variables, and the subgroup of age≤25 years was determined as reference. TSH was included as covariate. This model indicated that the OR of HUA was clearly negatively associated with an increase in age for males, except for the eldest subgroup of age>75 years, which displayed that the risk of HUA generally decreased accompanied with aging after adjustment for TSH values. On the contrary, the risk of HUA was clearly positively associated with an increasing age over 55 years in females.

## 4. Discussion

The current study revealed that both gender and age had a substantial impact on the association between thyroid function and HUA in the large-scale, single-center Chinese population. We found that the risk of HUA was positively associated with an elevation in TSH levels in both genders. Males with a high TSH value were more vulnerable to suffer significant risk of HUA. Aging was a significant risk factor for HUA, particularly in older females after adjusting for TSH.

This current study showed that the overall prevalence of HUA was significantly higher in males than in females (Table 1). Increasing risks of HUA in females were found positively along with aging. Conversely, males showed a reduced trend in HUA prevalence negatively with aging (Figure 3). Prevalence of HUA decreased until the age of midlife (36–45 years) and increased from the period of menopause in women. Our study confirmed that age is an independent risk factor of HUA after adjusting for TSH value, especially in the old-aged female (Table 4). The gender discrepancy in HUA prevalence is narrowing from the turning point of menopause. Consistent to previous studies [12–14], Kim et al. described the age-specific prevalence of HUA showed a U-shaped relationship with age in both sexes in Korean population, and it was least prevalent in women aged 40–49 years and men aged 70–79 years [12]. The critical mechanisms may be as follows. First, estrogen increases renal urate clearance and lowers tubular urate postsecretory reabsorption in females before menopause [15]. Yet, after menopause, with the decline of ovarian function, leading to the declining of circulating sex hormones, estrogens elevate SUA in older women [16–18]. In addition, the SUA level will be decreased after hormone replacement therapy in postmenopausal women with HUA [19, 20], further confirming the estrogens' protective effect of UA.

TSH is the most sensitive marker of thyroid function. Our results concluded that the incidence of thyroid dysfunction and TSH levels are higher in women than in men. These results were consistent with the findings of other epidemiological studies [21–23]. Some previous literature proposed that serum TSH levels can be strongly affected by age, gender, genetics, thyroid autoantibodies, iodine intake, and thyroid-autoimmune diseases [24–26]. In this study, due to lacking data of concomitant diseases and diet lifestyle of the population, we only found that females had more proportion of TPOAb and TgAb. In addition, estrogen may involve in the process; it may modulate the effect of excessive iodine on thyroid tissue and the estrogen receptor mediated increase in oxidative stress in women and may account for higher TSH value and higher prevalence of thyroid dysfunction in females [27]. In line with other studies [23], we also demonstrated that the median TSH value increases with aging; this could be explained by a normal physiologic response to compensate for the decrease in TSH biological activity due to age-related changes in TSH glycosylation with aging [28, 29]. Also, we found that TSH and TT4 were significantly lower, while TT3 was higher in males than in females. T4 is only produced by the thyroid gland, while T3 can be produced in the thyroid and in many other tissues from T4 deiodination. Since almost all T4 and T3 in serum are bound to several serum proteins, serum alterations in the concentration of binding proteins can have a significant effect on T4 and T3 levels and their partial metabolism, while we did not have the information of concomitant diseases, hormone therapy, and nutritional status between gender that could affect binding proteins, so we need more high-quality research to confirm and clarify this result.

Our main finding was the positive relationship between TSH value and HUA after binary logistic regression analysis, strongly evident in the high TSH group of males. In other words, males with a high TSH value were vulnerable to suffer a significant risk of HUA irrespective of age. In line with our findings, Zhang et al. demonstrated that mild hypothyroidism is a risk factor for HUA in males, while it is not in females. The underlying mechanisms between thyroid function and HUA are not fully understood, and we try to explain this in the following points. First, studies had mentioned that estrogen contributes to a lower SUA level by promoting UA degradation and excretion, but its protective effect against TSH developing HUA in women was underlined. Second, it is well established that e-GFR has a negatively significant relationship with the SUA level. We found that the lower e-GFR in males is lower than that in females. Previous studies demonstrated that high TSH levels were associated with e-GFR and readily reversible by thyroxin replacement therapy; this can be another possible reason [30–32].

To our knowledge, this is the first investigation which confirmed that the impact of increased TSH level on SUA from the perspective of gender and age. Future population-based and cross-sectional studies are needed to confirm the relationship between thyroid function and HUA.

## 5. Limitations

There are some limitations to our study that should be noted. First, this study was an observational design that may suffer from potential selection and measurement biases. Second, while the study provided a large sample size, the research was limited to a single center and single ethnicity, which may limit the reliability and generalizability of our findings; these results need to be validated or tested among other center or ethnicities. Third, detailed data about thyroid function indicators including free hormones (FT3 and FT4) were not collected, and follow-up data are not available, so that we failed to assess the potential impacts, so further studies are encouraged and needed.

## Figures and Tables

**Table 1:** Population characteristics based on different genders.

Variables	Total (*n* = 19013)	Males (*n* = 10563)	Females (*n* = 8450)	*P* value
Age (years)	47.50 ± 14.51	48.43 ± 14.44	46.35 ± 14.52	<0.001
BMI (kg/m^2^)	24.42 ± 3.51	25.66 ± 3.15	22.83 ± 3.30	<0.001
WC (cm)	84.54 ± 10.75	90.17 ± 8.46	77.22 ± 8.83	<0.001
SBP (mm·Hg)	124.39 ± 19.29	127.98 ± 17.78	119.83 ± 20.15	<0.001
DBP (mm·Hg)	76.94 ± 11.08	79.72 ± 10.77	73.40 ± 10.45	<0.001
TSH (*u*IU/mL)	2.33 ± 2.38	2.18 ± 2.48	2.52 ± 2.25	<0.001
TT3 (pmol/L)	1.12 ± 0.21	1.15 ± 0.22	1.08 ± 0.20	<0.001
TT4 (pmol/L)	7.96 ± 1.52	7.93 ± 1.52	8.02 ± 1.54	0.029
TC (mmol/L)	4.83 ± 0.94	4.78 ± 0.94	4.89 ± 0.93	<0.001
TG (mmol/L)	1.19 (0.82–1.77)	1.38 (0.97–2.04)	0.98 (0.71–1.44)	<0.001
LDL-C (mmol/L)	3.09 ± 0.85	3.11 ± 0.84	3.06 ± 0.86	<0.001
hsCRP (mg/dL)	0.62 (0.13–1.53)	0.70 (0.20–1.74)	0.51 (0.04–1.34)	<0.001
UA (*u*mol/L)	328.13 ± 86.32	372.81 ± 76.93	272.28 ± 61.27	<0.001
Cr (*u*mol/L)	65.50 ± 17.47	74.50 ± 16.93	54.25 ± 10.03	<0.001
BUN (mmol/L)	5.01 ± 1.36	5.34 ± 1.38	4.60 ± 1.21	<0.001
FG (mmol/L)	5.59 ± 1.34	5.76 ± 1.50	5.38 ± 1.08	<0.001
HbA1c (%)	5.71 ± 0.75	5.77 ± 0.81	5.63 ± 0.66	<0.001
ALT (*u*/l)	22.44 ± 17.12	26.40 ± 18.45	17.49 ± 13.80	<0.001
AST (*u*/l)	22.45 ± 11.65	23.66 ± 11.51	20.76 ± 11.63	<0.001
e-GFR (ml/min/1.73 m^2^)	105.38 ± 15.46	102.67 ± 15.06	108.76 ± 15.29	<0.001
TPOAb positive, *n* (%)	693 (3.6)	255 (2.4)	438 (5.2)	<0.001
TGAb positive, *n* (%)	783 (4.1)	232 (2.2)	551 (6.5)	<0.001

Thyroid function, *n* (%)				
Euthyroidism	18224 (95.9)	10240 (53.9)	7984 (42.0)	<0.001
Hypothyroidism^†^	644 (3.4)	264 (1.4)	380 (2.0)	<0.001
Hyperthyroidism^†^	145 (0.7)	59 (0.3)	86 (0.4)	<0.001

HUA, *n* (%)	3304 (17.4)	2640 (25.0)	664 (7.9)	<0.001

BMI, body mass index; WC, waist circumference; SBP, systolic blood pressure; DBP, diastolic blood pressure; TSH, thyrotropin; TT3, total triiodothyronine; TT4, total thyroxine; TC, total cholesterol; TG, triglyceride s; LDL-C, low-density lipoprotein cholesterol; hsCRP, high-sensitivity C-reactive protein; UA, uric acid; Cr, creatinine; BUN, blood urea nitrogen; FG, fasting glucose; HbA1c, glycosylated hemoglobin A1c; ALT, alanine transaminase; AST, aspartate aminotransferase; e-GFR, estimated glomerular filtration rate; TPOAb, thyroid peroxidase autoantibody; TGAb, thyroglobulin autoantibody; HUA, hyperuricemia.

**Table 2:** Incidence of hypothyroidism and hyperthyroidism on different genders.

	Incidence (and case number count) in different age subgroups, years							
	Age ≤ 25	25 ≤ age ≤ 35	35 age ≤ 45	45 ≤ age ≤ 55	55 ≤ age ≤ 65	65 ≤ age ≤ 75	Age ≤ 75	Total
Male								
Euthyroidism	173 (96.6%)	2014 (98.4%)	2572 (98.2%)	2728 97.6%)	1488 (94.7%)	709 (94.3%)	556 (92.5%)	10240 (96.9%)
Hypothyroidism^†^	5 (2.8%)	`18 (0.9%)	34 (1.3%)	56 (2.0%)	69 (4.4%)	40 (5.3%)	42 (7.0%)	264 (2.5%)
Hyperthyroidism^†^	1 (0.6%)	15 (0.7%)	13 (0.5%)	10 (0.4%)	14 (0.9%)	3 (0.4%)	3 (0.5%)	59 (0.6%)

Female								
Euthyroidism	284 (96.3%)	1978 (96.4%)	1848 (96.0%)	1103 (89.5%)	1987 (95.7%)	464 (91.9%)	320 (87.7%)	7984 (94.5%)
Hypothyroidism^†^	7 (2.3%)	53 (2.6%)	54 (2.8%)	111 (9.0%)	78 (3.8%)	37 (7.3%)	40 (11.0%)	380 (4.5%)
Hyperthyroidism^†^	4 (1.4%)	20 (1.0%)	22 (1.2%)	19 (1.5%)	12 (0.5%)	4 (0.8%)	5 (1.3%)	86 (1.0%)

Chi-square value^‡^								
Hypothyroidism^†^	0.080	17.488^*∗∗*^	13.287^*∗∗*^	38.465^*∗∗*^	6.202^*∗*^	2.117	4.609^*∗*^	57.254^*∗∗*^
Hyperthyroidism^†^	0.678	0.711	6.075^*∗*^	5.974^*∗*^	0.096	0.843	2.096	13.080^*∗∗*^
Total	0.751	18.289^*∗∗*^	19.587^*∗∗*^	44.887^*∗∗*^	6.336^*∗*^	3.013	6.883^*∗*^	71.251^*∗∗*^

^*∗*^ *P* < 0.05. ^*∗∗*^*P* < 0.01. ^†^Hypothyroidism defined as TSH >5.5 mIU/mL and hyperthyroidism defined as TSH <0.35 mIU/mL. ^‡^Comparing the incidence of hypothyroidism and/or hyperthyroidism between males and females by the chi-squared method.

**Table 3:** Pearson bivariate correlations between UA and other variables.

Variables	Male (correlation coefficients)	Female (correlation coefficients)
Age	−0.183^*∗∗*^	0.164^*∗∗*^
BMI	0.268^*∗∗*^	0.316^*∗∗*^
WC	0.232^*∗∗*^	0.305^*∗∗*^
SBP	0.016	0.213^*∗∗*^
DBP	0.126^*∗∗*^	0.177^*∗∗*^
TSH	0.027^*∗∗*^	0.045^*∗∗*^
TT3	0.003	0.069^*∗∗*^
TT4	−0.040^*∗*^	0.042^*∗*^
TC	0.120^*∗∗*^	0.122^*∗∗*^
TG	0.211^*∗∗*^	0.277^*∗∗*^
LDL-C	0.082^*∗∗*^	0.146^*∗∗*^
Cr	0.167^*∗∗*^	0.274^*∗∗*^
BUN	0.034^*∗∗*^	0.220^*∗∗*^
FG	−0.094^*∗∗*^	0.143^*∗∗*^
HbA1	−0.105^*∗∗*^	0.160^*∗∗*^
e-GFR	−0.026^*∗∗*^	0.274^*∗∗*^

BMI, body mass index; WC, waist circumference; SBP, systolic blood pressure; DBP, diastolic blood pressure; TSH, thyrotropin; TT3, total triiodothyronine; TT4, total thyroxine; TC, total cholesterol; TG, triglycerides; LDL-C, low-density lipoprotein cholesterol; Cr, creatinine; BUN, blood urea nitrogen; FG, fasting glucose; HbA1c, glycosylated hemoglobin A1c; e-GFR, estimated glomerular filtration rate. ^*∗*^*P* < 0.05, ^*∗∗*^*P* < 0.01.

**Table 4:** The risks of developing HUA in different genders.

	Males		Females	
	Parameter values	OR (95% CI)	Parameter values	OR (95% CI)
TSH functions^†^	(uIU/mL)		(uIU/mL)	< 0.001
Perfect function	0.35TSH ≤ 2.5 (reference)	—	0.35TSH ≤ 2.5 (reference)	—
Normal function	2.5TSH ≤ 5.5	1.229 (1.107–1.363)	2.5TSH ≤ 5.5	1.198 (1.011–1.420)
Mild hypothyroidism	5.5TSH ≤ 10.0	1.370 (1.006–1.866)	5.5TSH ≤ 10.0	1.256 (0.858–1.838)
Overt hypothyroidism	TSH > 10.0	1.408 (0.704–2.817)	TSH > 10.0	1.458 (0.685–3.105)
Hyperthyroidism	TSH ≤ 0.35	0.634 (0.319–1.259)	TSH ≤ 0.35	0.770 (0.308–1.920)

TSH quartiles^†^	(uIU/mL)		(uIU/mL)	
Quartile 1	TSH ≤ 1.32 (reference)		TSH ≤ 1.48 (reference)	
Quartile 2	1.32TSH ≤ 1.82	1.076 (0.948–1.222)	1.48TSH ≤ 2.10	0.887 (0.702–1.121)
Quartile 3	1.82TSH ≤ 2.53	1.108 (0.976–1.259)	2.10TSH ≤ 3.01	0.976 (0.777–1.225)
Quartile 4	TSH > 2.53	1.322 (1.166–1.500)	TSH > 3.01	1.119 (0.899–1.392)

Age subgroups†	(Years)		(Years)	
Age subgroup 1	15 < age ≤ 25 (reference)	—	15 < age ≤ 25 (reference)	—
Age subgroup 2	25 age ≤ 35	0.671 (0.491–0.916)	25age ≤ 35	0.879 (0.556–1.392)
Age subgroup 3	35 age ≤ 45	0.582 (0.427–0.794)	35age ≤ 45	0.538 (0.333–0.868)
Age subgroup 4	45 age ≤ 55	0.439 (0.322–0.599)	45age ≤ 55	0.836 (0.529–1.324)
Age subgroup 5	55 age ≤ 65	0.331 (0.239–0.457)	55age ≤ 65	1.447 (0.911–2.299)
Age subgroup 6	65 < age ≤ 75	0.253 (0.176–0.362)	65age ≤ 75	2.310 (1.420–3.758)
Age subgroup 7	75 < age ≤ 97	0.347 (0.242–0.498)	75 < age ≤ 97	2.432 (1.467–4.031)

CI, confidence interval; TSH, thyroid stimulation hormone. ^†^Logistic regression model included age, TSH as covariates.

## Data Availability

The data used to support the findings of this study are available from the corresponding author upon request.
